# PROACTING: predicting pathological complete response to neoadjuvant chemotherapy in breast cancer from routine diagnostic histopathology biopsies with deep learning

**DOI:** 10.1186/s13058-023-01726-0

**Published:** 2023-11-13

**Authors:** Witali Aswolinskiy, Enrico Munari, Hugo M. Horlings, Lennart Mulder, Giuseppe Bogina, Joyce Sanders, Yat-Hee Liu, Alexandra W. van den Belt-Dusebout, Leslie Tessier, Maschenka Balkenhol, Michelle Stegeman, Jeffrey Hoven, Jelle Wesseling, Jeroen van der Laak, Esther H. Lips, Francesco Ciompi

**Affiliations:** 1 Department of Pathology, Radboud University Medical Center, Nijmegen, The Netherlands; 2 Department of Molecular and Translational Medicine, University of Brescia, Brescia, Italy; 3 The Netherlands Cancer Institute (NKI), Amsterdam, The Netherlands; 4 Pathology Unit, IRCCS Sacro Cuore Don Calabria Hospital, Negrar di Valpolicella, Verona, Italy; 5 Center for Integrated Oncology (Institut du cancer de l’Ouest), Angers, France; 6 Leiden University Medical Center, Leiden, The Netherlands

**Keywords:** Neoadjuvant chemotherapy, Pathological complete response, Computational biomarker

## Abstract

**Background:**

Invasive breast cancer patients are increasingly being treated with neoadjuvant chemotherapy; however, only a fraction of the patients respond to it completely. To prevent overtreatment, there is an urgent need for biomarkers to predict treatment response before administering the therapy.

**Methods:**

In this retrospective study, we developed hypothesis-driven interpretable biomarkers based on deep learning, to predict the pathological complete response (pCR, i.e., the absence of tumor cells in the surgical resection specimens) to neoadjuvant chemotherapy solely using digital pathology H&E images of pre-treatment breast biopsies. Our approach consists of two steps: First, we use deep learning to characterize aspects of the tumor micro-environment by detecting mitoses and segmenting tissue into several morphology compartments including tumor, lymphocytes and stroma. Second, we derive computational biomarkers from the segmentation and detection output to encode slide-level relationships of components of the tumor microenvironment, such as tumor and mitoses, stroma, and tumor infiltrating lymphocytes (TILs).

**Results:**

We developed and evaluated our method on slides from *n* = 721 patients from three European medical centers with triple-negative and Luminal B breast cancers and performed external independent validation on *n* = 126 patients from a public dataset. We report the predictive value of the investigated biomarkers for predicting pCR with areas under the receiver operating characteristic curve between 0.66 and 0.88 across the tested cohorts.

**Conclusion:**

The proposed computational biomarkers predict pCR, but will require more evaluation and finetuning for clinical application. Our results further corroborate the potential role of deep learning to automate TILs quantification, and their predictive value in breast cancer neoadjuvant treatment planning, along with automated mitoses quantification. We made our method publicly available to extract segmentation-based biomarkers for research purposes.

**Supplementary Information:**

The online version contains supplementary material available at 10.1186/s13058-023-01726-0.

## Background

Invasive breast cancer (IBC) is increasingly being treated with chemotherapy administered *prior* to breast cancer surgery [1]. This *neoadjuvant* chemotherapy (NAC) is intended to reduce the tumor load and may result in the pathological complete response (pCR), i.e., the absence of visible tumor cells in the surgery resections. Studies have shown that pCR is associated with event-free survival and recurrence-free survival [2]. However, only a fraction of treated patients responds to the treatment, with response rates that vary with the molecular subtypes of breast cancer. About 40% of patients with triple-negative breast cancers (TNBC) will achieve pCR, whereas the response rate for Luminal B breast cancer patients is only about 15% [3, 4].

Administering NAC is a process that lasts for several weeks, has side effects and de-facto postpones the surgery while the tumor may progress locally and systemically if the patient does not respond. This shows the urgent need for predicting whether treating a patient with NAC will result in pCR, to optimally plan the treatment strategy.

Several studies have shown the correlation between visual assessment of components of the tumor micro-environment (TME) and favorable NAC response and survival outcomes. One example is the assessment of stromal tumor-infiltrating lymphocytes (TILs), often quantified on hematoxylin and eosin (H&E) stained slides following the recommendations from the International TILs Working Group [5–8]. Although effective, visual TILs assessment is hampered by potential “pitfalls” such as the presence of, e.g., ischemic tumor cells, small tumor nuclei, and fixation artifacts [9] and requires the mental exclusion of regions such as benign tissue and in situ lesions.

Another feature of the TME with predictive value for NAC response is the tumor proliferation score [10], assessed based on Ki67 immunohistochemistry staining as the percentage of tumor cells with positive nuclear staining [11, 12]. However, Ki67 staining may not be routinely available and introduces additional costs compared to standard diagnostic H&E staining.

To automate biomarker quantification, in recent years, researchers have started focusing on deep learning with convolutional neural networks (CNN) to learn directly from raw image data [13, 14]. In the context of deep learning for computational biomarkers such as TIL scoring in breast cancer, several approaches have been recently proposed based on CNNs [15–19]. In most cases, these approaches segment the tissue into tumor, stroma and lymphocyte compartments and compute the TIL-score based on the compartment ratios. Although these approaches would be an effective base for biomarker development, they were evaluated only on surgery resections, not diagnostic biopsies, and their predictive value for NAC response was not investigated.

For pCR prediction from H&E stained biopsies, several studies have been carried out, focusing either on the tumor-epithelium [20, 21] or tumor-associated stroma [22]. Some approaches also combined information in H&E and immunohistochemistry (IHC) [23, 24]. Another recent approach increased the sample size via federated multi-instance learning [25]. While these studies showed promising results, either their validation is limited to small datasets or the learned scores lack morphological interpretability.

In this work, we focus on two specific breast cancer subtypes, namely triple-negative (TNBC: HR−, HER2−) and Luminal B (HR+, HER2−, grade 2/3) invasive breast cancers. We investigate aspects of the TME by proposing *hypothesis-driven* interpretable computational biomarkers based on relations of different tissue morphologies. Specifically, we formulate four biomarkers grounded on largely accessible morphological features: the ratio of lymphocytes to tumor in the slide (LTR), the ratio of (inflamed) tumor close to lymphocytes to the overall tumor amount (ITR), the computational tumor infiltrating lymphocytes score (cTILs) and the mitotic rate (MTR) as the number of detected mitoses within tumor regions divided by the tumor area. For this purpose, we propose a modular two-step approach where we first use neural networks to quantify tissue compartments and detect mitoses, and then use these outputs to encode biomarkers. To the best of our knowledge, this is the first time that H&E-based mitotic count is considered in the design of predictive biomarkers for NAC response. We validate performance of the biomarkers for predicting pCR response to NAC and compare the predictive performance of the computational biomarkers with the visually assessed TILs-score (vTILs). We refer to the proposed biomarkers as 'PROACTING' (PRedicting neOAdjuvant Chemotherapy Treatment response with deep learnING).

## Materials and methods

In this section, we first introduce the data used for the development and the validation of the multiple parts of this study, and then, we introduce the methodology used.

### Clinical focus and definitions

#### Breast cancer subtypes

The primary focus of our study is on triple-negative (TNBC: HR−, HER2−) and ‘surrogate’ Luminal B (HR+, HER2−, grade 2/3) invasive breast cancers. As gene expression data and Ki67 were not available in our cohorts, we discriminated between ‘surrogate’ Luminal A and B based on the grade; this definition has been shown to provide chemotherapy benefit [27]. For the sake of compactness, in the rest of the paper, we will refer to “surrogate” Luminal B as simply Luminal B.

Additionally, we evaluated the developed biomarkers on an external public dataset from the IMPRESS study [24], which contains both TNBC and HER2+ cases.

#### Definition of pCR

We define here the pathological complete response to NAC as the absence of invasive cancer in the breast only (ypT0/is [26]). Focusing on breast only provides the closest readout when biopsies from the primary tumor in the breast are analyzed, yet providing sufficient predictive value to support treatment planning.

### Method overview

**Fig. 1:**
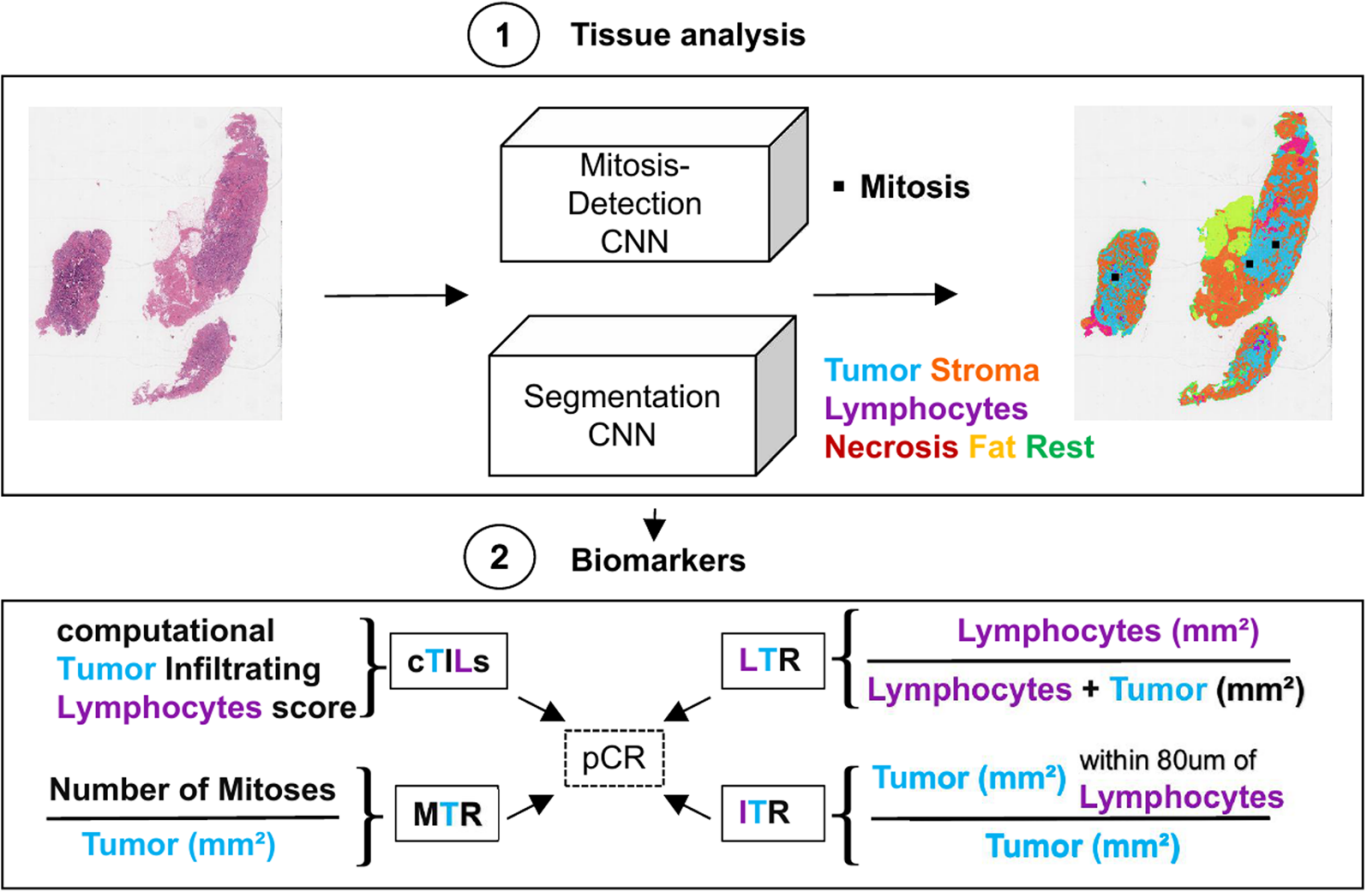
Method overview: (1) Segment slides into different tissue types and detect mitoses. (2) Compute biomarkers from the segmentation prediction of tumor, stroma and lymphocytes and detected mitoses within tumor regions. LTR: lymphocyte-tumor ratio, cTILs: computational tumor infiltrating lymphocytes score, ITR: inflamed tumor ratio (proportion of tumor close to lymphocytes), MTR: mitoses-tumor ratio

Our approach consists of two parts, visualized in Fig. 1. First, we trained a CNN to segment the slides into the classes tumor, stroma, lymphocytes, necrosis, fat and rest. We also used an existing CNN model for mitosis detection developed by Tellez et al., previously validated in clinical studies [28, 29]. The output of this deep learning pipeline for a slide is a segmentation mask for the six classes and the coordinates of detected mitoses in the tumor regions. Second, we derived biomarkers from the tissue segmentation and mitoses detections and assessed their predictive value for pCR. In this section, we first introduce the used data and then the developed methods.

### Data

**Fig. 2:**
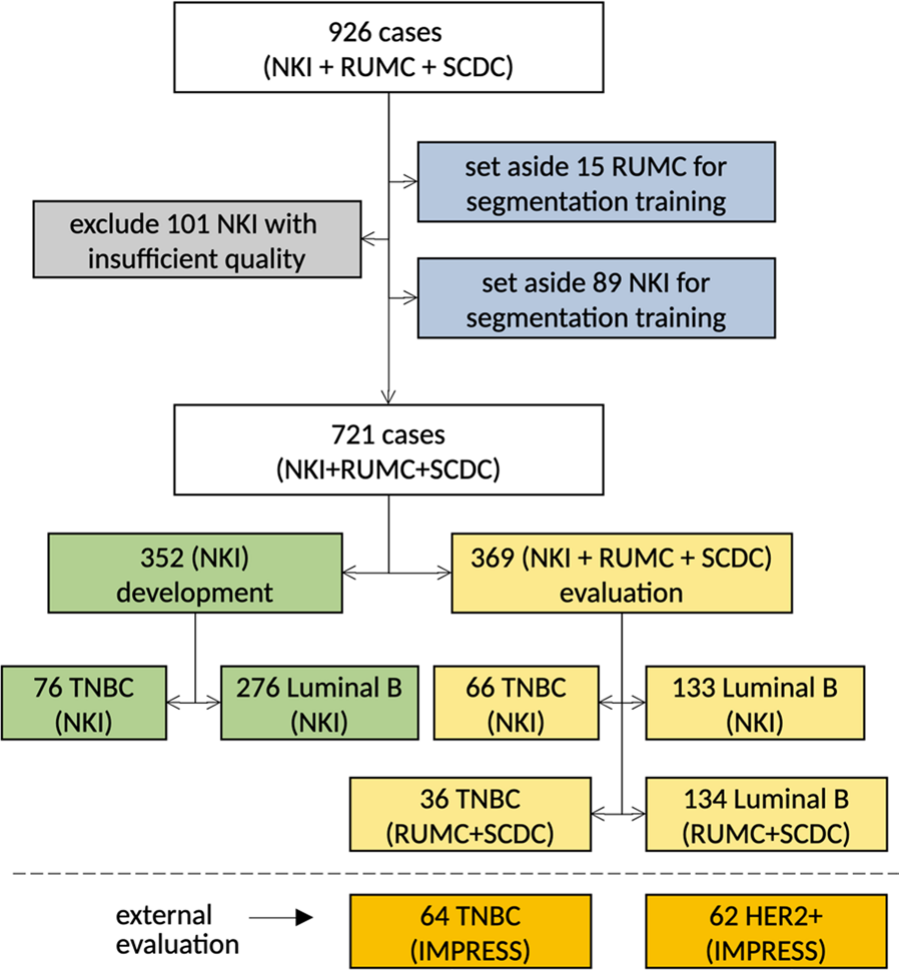
Biomarker development and evaluation data: visualization of the data split per type (TNBC, Luminal B), center (NKI, RUMC+SCDC, IMPRESS) and data subset (development, evaluation), starting from the exclusion of cases due to quality (in gray) and for training of the segmentation model (in blue, part of ${\text{dev}}_{\rm seg}^{\rm train}$) to the definition of the development (in green, ${\text{dev}}_{\rm bm}$) and evaluation (in yellow, ${\text{val}}_{\rm int}$) datasets. Shown is also the additional IMPRESS [24] evaluation data (in orange, ${\text{val}}_{\rm ext}$). Not included is the additional data for segmentation model training

In this section, we first introduce the cohorts included in this study as well as the case inclusion and exclusion criteria. Based on that, we then describe the datasets used in the multiple phases of development and evaluation of the proposed work. In particular, we have defined (1) a dataset for the training of our segmentation algorithm on H&E slides; (2) a dataset for the development and tuning of the computational biomarkers; (3) an internal evaluation set; and (4) an external independent evaluation set. The data split is visualized in Fig. 2.

*Cohorts* For model development and internal evaluation, we collected 926 cases from three European centers: 741 from the Netherlands Cancer Institute (NKI, Amsterdam, the Netherlands), 123 from the Radboud University Medical Center (RUMC, Nijmegen, the Netherlands) and 62 from the IRCCS Sacro Cuore Don Calabria Hospital (SCDC, Verona, Italy). All slides are diagnostic biopsies stained with H&E, extracted via core-needle procedure (before NAC). For NKI TNBC and the RUMC cases, multiple slides per case are available while the other cohorts have only one slide per case. In all cases, cohorts included both cases of Luminal B (defined as HR+, HER2−, grade 2/3) and triple-negative breast cancers (TNBC, defined as HR−, HER2−). For all cohorts, information about the NAC response was available; additional available clinical information (after exclusion) is listed in Tables 1 and 2.

**Table 1:** Clinical information for the TNBC cohorts per center (NKI, RUMC and SCDC)

	NKI	%	RUMC	%	SCDC	%
Cases	142	100	21	100	15	100
Slides	172	100	67	100	15	100
Response						
pCR = 0	72	51	13	62	8	53
pCR = 1	70	49	8	38	7	47
Age						
Age ≤ 50	91	64	14	67	7	47
Age > 50	51	36	7	33	8	53
Grade						
Grade = 2	34	24	4	19	2	13
Grade = 3	90	63	12	57	13	87
Unknown	18	13	5	24	–	–
T stage						
T1/2	104	73	–	–	–	–
T3/4	34	24	–	–	–	–
Unknown	4	3	21	100	15	100
N stage						
N0	71	50	–	–	–	–
N1	70	49	–	–	–	–
Unknown	1	1	21	100	15	100
Histology						
IDC	110	77	–	–	14	93
ILC	5	4	–	–	–	–
Invasive mixed	2	1	–	–	–	–
Other	5	4	–	–	1	7
Unknown	20	14	21	100	–	–

**Table 2:** Clinical information for the Luminal B cohorts per center (NKI, RUMC and SCDC)

	NKI	%	RUMC	%	SCDC	%
Cases	409	100	87	100	47	100
Slides	409	100	279	100	47	100
Response						
pcr = 0	372	91	83	95	45	96
pcr = 1	37	9	4	5	2	4
Age						
Age ≤ 50	216	53	55	63	24	51
Age > 50	193	47	32	37	23	49
Grade						
Grade = 2	289	70	52	60	33	70
Grade = 3	113	28	35	40	14	30
Unknown	7	2	–	–	–	–
T stage						
T1/2	288	70	–	–	–	–
T3/4	112	28	–	–	–	–
Unknown	9	2	87	100	47	100
N stage						
N0	121	30	–	–	–	–
N1	279	68	–	–	–	–
Unknown	9	2	87	100	47	100
Histology						
IDC	321	78	–	–	43	92
ILC	74	18	–	–	3	6
Mixed	7	2	–	–		
Other	4	1	–	–	1	2
Unknown	3	1	87	100	–	–

Slides from NKI were obtained from retrospective studies and include old glass slides. Therefore, after digitization, slides were visually inspected by pathologists, who excluded 101 slides with washed-out staining or too few tumor cells. Slides from SCDC were checked by pathologists at the time of inclusion in this study, and the RUMC slides were scanned for the purpose of this study and visually checked for quality before and after scanning, resulting in no exclusion due to quality issues. All slides were digitized in the originating clinical center using multiple scanners. The NKI TNBC slides were scanned with an Aperio AT2 (Leica Biosystems) at 40X, the NKI Luminal B slides with a PANNORAMIC 1000 (3DHISTECH) scanner at 40X; the RUMC slides with a 3DHistech Pannoramic 1000 scanner at 40×; the slides from SCDC with a Ventana DP 200 slide scanner at 20× magnification.

For external evaluation, we used data from the public dataset recently published by Huang et al. [24] (IMPRESS). This cohort contains 64 TNBC cases and 62 HER2+ cases. The slides contain core-needle biopsies of breast cancer tissue samples, scanned at 20× magnification with a Hamamatsu scanner.

**Fig. 3:**
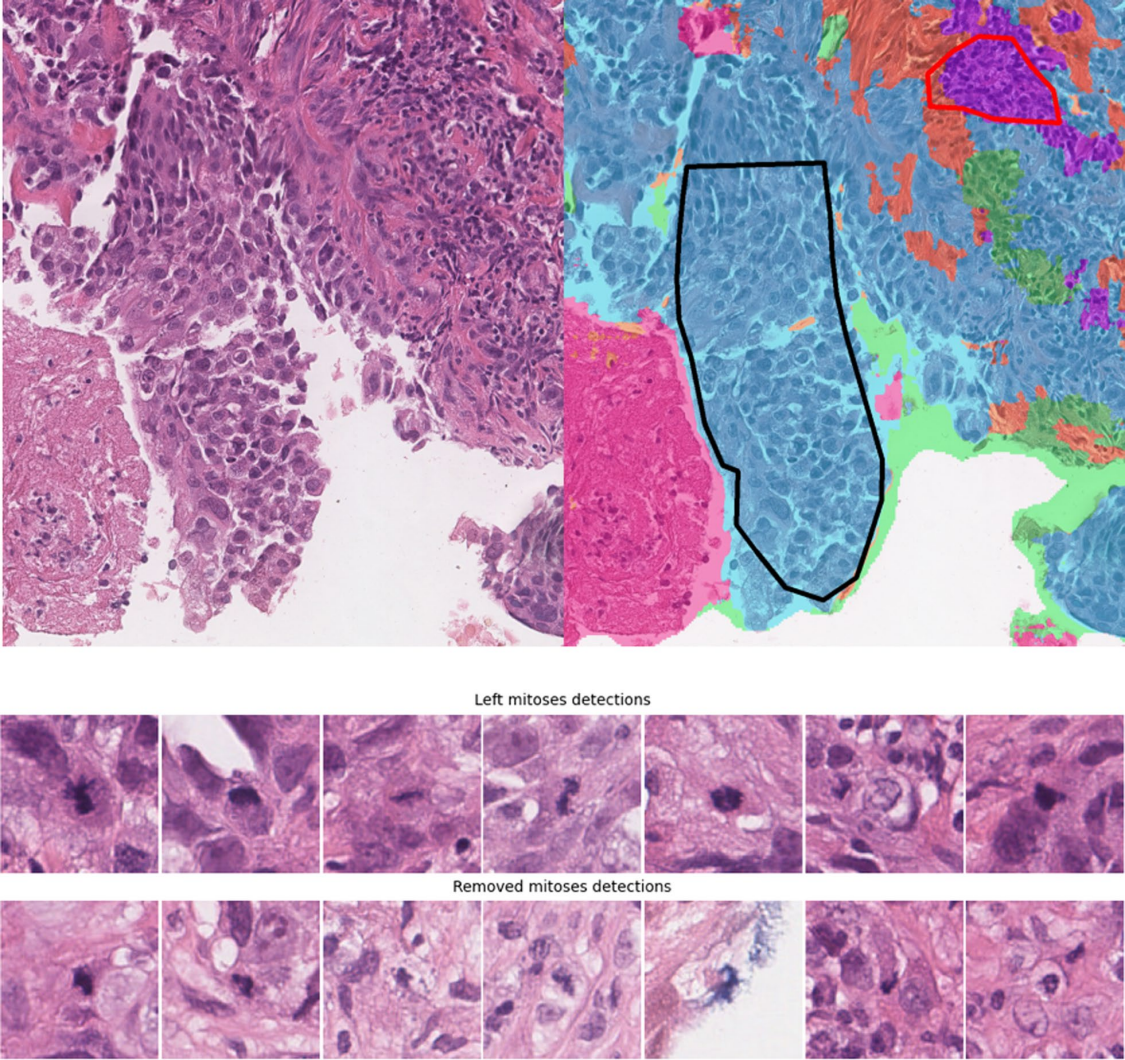
Segmentation and detection examples. On the top left is an example from a test slide with the segmentation overlay on the right. Predicted tumor is hued blue, necrosis magenta, lymphocytes purple, stroma orange and the rest green. The drawn polygons are the tissue annotations (red: Lymphocytes, black: Tumor). The slides were annotated using ASAP(https://github.com/computationalpathologygroup/ASAP). On the bottom are examples of kept (top) and filtered out (bottom) mitoses detections

*Development set for segmentation algorithm* To train the multi-class tissue segmentation model, we assembled manually annotated cases from three different types of datasets, to form a development dataset. First, we used *n* = 110 biopsy cases from the NKI and the RUMC cohorts assembled within this project. In detail, we included breast biopsies from 89 NKI cases with *n* = 95 slides (82 TNBC, 13 Luminal B, as some cases have multiple slides), and from 15 RUMC cases, where one slide per patient was selected. Since these slides were used for training the segmentation model, they were excluded from the biomarker evaluation. Research assistants, instructed and supervised by pathologists, annotated small tissue regions on these slides as tumor, stroma, lymphocytes, necrosis, fatty tissue or rest/normal. Differentiating between tumor, stroma and lymphocytes is essential for the characterization of features of the TME, such as assessment of TILs, whereas the other classes were added for a more comprehensive tissue differentiation. An example of two annotations is shown in Fig. 3. Second, we included *n* = 92 slides from the public Breast Cancer Semantic Segmentation study (BCSS, [30]), with annotations for TNBC resections from TCGA [31]). These slides were densely annotated in regions of interest (i.e., all pixels in the ROI were labeled) with 18 different tissue types, which we mapped into the six targeted classes for consistency with the rest of the data.

Third, we included 73 slides from a RUMC cohort used in previous work to develop the “HookNet” model [32]. This dataset consisted of surgical resection slides which were manually annotated with sparse annotations of six classes of multiple tissue types.

Overall, 275 slides (165 resections, 110 biopsies) were used for model training, which we refer to as ${\text{dev}}_{\rm seg}^{\rm train}$, and 74 slides (59 resections from the BCSS dataset and 15 biopsies from the NKI TNBC dataset) were used as test set to assess performance of the segmentation model, which we refer to as ${\text{dev}}_{\rm seg}^{\rm test}$.

*Development set for computational biomarkers* We used data from 352 NKI cases (76 TNBC, 276 Luminal B) for the development and fine-tuning of computational biomarkers. Clinical and outcome data in terms of pCR were made available by the NKI. We used these data to design our computational biomarkers and fine-tune their parameters, e.g., choosing thresholds to maximize pCR prediction performance. We refer to this set as the ${\text{dev}}_{\rm bm}$ dataset. It includes 15 slides with manual tissue annotations, which are also part of ${\text{dev}}_{\rm seg}^{\rm test}$.

*Internal evaluation set.* We defined an internal evaluation set that contained 369 cases from NKI (66 TNBC, 133 Luminal B) and a combination of RUMC and SCDC cases, providing 170 cases in total (36 TNBC, 134 Luminal B). These cases were not used in any learning procedure, and the models’ predictions on them were evaluated externally by statisticians involved in this project only at the end of the fine-tuning phase of the computational biomarkers. We refer to this set as the ${\text{val}}_{\rm int}$ dataset.

*External evaluation set* We also considered an external public dataset of breast cancer biopsies, recently published by Huang et al. [24] (IMPRESS). This cohort contains 64 TNBC cases and 62 HER2+ cases stained with H&E. Although HER2+ was not a subtype explicitly considered in the learning phase of our method, given the general applicability of the proposed PROACTING biomarkers, we validated their predictive value on this subtype as well. We refer to this set as the ${\text{val}}_{\rm ext}$ dataset.

### Deep learning for tissue segmentation and mitosis detection

As the computer model for tissue segmentation, we chose U-Net [33], a CNN architecture for medical image segmentation. The details of the model and its hyperparameters are described in Additional file 1: Section S1.1. At test-time, every slide was pre-processed to exclude background and out-of-focus regions using a network that was previously developed and validated by Bándi et al. [34], therefore only producing a segmentation output for pixels belonging to the biopsy tissue.

The mitosis detection network had been previously presented by Tellez et al. [28] and was used off-the-shelf in this work. In brief, the network predicts the location of mitotic figures across the entire H&E slide. Since the network operates at 40× magnification, to apply the network to the SCDC dataset scanned at 20x, we first upsampled the slides to 40× using bilinear interpolation. Initial visual inspection of the mitoses predictions for slides from the ${\text{dev}}_{\rm bm}$ set showed the presence of false positive detections outside of tumor regions. To address this issue, we combined the mitosis detection with the multi-class segmentation results and only kept mitoses surrounded by tumor at least 20 μm wide. This distance was determined empirically.

### Computational biomarkers

The segmentation maps and mitosis detections from the deep learning pipeline allow to define biomarkers based on different counts and ratios of the predicted tissues. Based on hypothesis on the role of tissue compartments in the TME, we designed four morphologically interpretable biomarkers, which we refer to as the PROACTING biomarkers: three related to TILs and one related to mitotic count. The hyper-parameters for the biomarkers, such as values for distances and thresholds, were tuned empirically on the ${\text{dev}}_{\rm bm}$ set to increase pCR prediction performance.

**Fig. 4:**
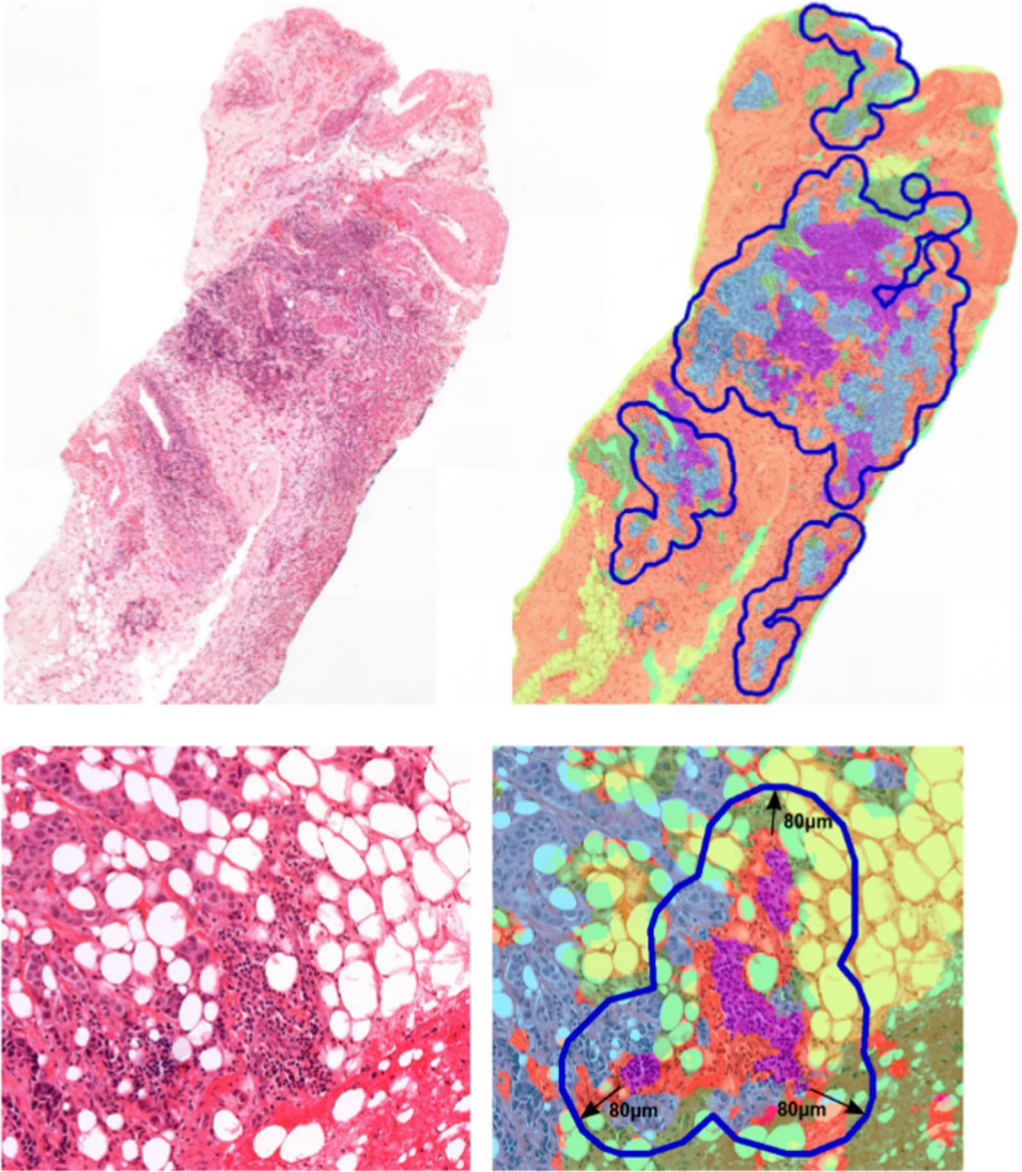
Visualization of the cTILs bulk (top) and the ITR radius (bottom) via blue polygons. In the overlays (right), tumor is hued blue, stroma orange, lymphocytes purple, necrosis magenta, fatty tissue yellow and the rest green

*Computational TILs* The biomarker cTILs (computational TILs) is aimed to emulate the visual estimation of stromal TILs as proposed by the International TILs Working Group [6]. To this end, the tumor bulk is determined by joining tumor regions within 100 μm clustering distance and creating an outlining envelope with a 50 μm margin around them. This is done via the morphological *closing* operation on the predicted tumor mask using a circular kernel with the clustering distance as radius. Then, the tumor mask is *dilated* by the margin distance (see Fig. 4 top). In the resulting tumor bulk, lymphocytes and stroma are counted:

1 $$\begin{aligned} {\text{cTILs}} = \frac{{\text{lymphocytes}} [{\text{mm}}^{2}]}{{\text{lymphocytes}}+{\text{stroma}} [{\text{mm}}^{2}]}. \end{aligned}$$

Tumor regions smaller than 0.1 mm^2^ were excluded from the tumor bulk formation to account for small wrong tumor predictions.

*Lymphocytes to tumor ratio* This biomarker measures the slide-global lymphocytes to tumor ratio (LTR):

2 $$\begin{aligned} {\text{LTR}} = \frac{{\text{lymphocytes}} [{\text{mm}}^{2}]}{{\text{lymphocytes}}+{\text{tumor}} [{\text{mm}}^{2}]}, \end{aligned}$$

where *lymphocytes* and *tumor* are the predicted area in mm$^2$ for the corresponding tissue type from all cores containing tumor predictions.

*Inflamed tumor ratio* The ‘inflamed’ tumor ratio biomarker (ITR) measures the ratio of tumor near lymphocytes to the overall tumor amount:

3 $$\begin{aligned} {\text{ITR}} = \frac{{\text{tumor within}}\, 80\,\upmu{\text{m}}\,{\text{of lymphocytes}} [{\text{mm}}^{2}]}{{\text{tumor}} [{\text{mm}}^{2}]} \end{aligned}$$

The value for the lymphocyte-tumor ‘interaction’ distance of 80 μm was chosen empirically. (An example is shown in Fig. 4 bottom.)

*Mitotic rate* The mitotic rate (MTR) measures the mitosis to tumor rate:

4 $$\begin{aligned} {\text{MTR}} = \frac{{\text{mitoses}}}{{\text{tumor}} [{\text{mm}}^{2}]}, \end{aligned}$$

where *mitoses* is the number of detected mitoses inside the segmented tumor regions and *tumor* the amount of predicted tumor in $mm^2$.

*Handling multiple biopsies and cores* Usually, a core needle biopsy procedure produces several cores. Only cores containing predicted tumor were considered for the biomarker computation, the rest was excluded. When multiple slides per case were present, the computational biomarkers were computed per case, as if all cores were present on a single slide.

### Visual TIL scoring

To compare our PROACTING computational biomarkers with visual TIL-scoring according to the recommendations of the TIL Working Group [6], we set up reader studies for two pathologists to score the NKI TNBC (scored by JS and EM) and the NKI Luminal B cohorts (scored by EM and HMH) using the web-based platforms SlideScore^1^ and CIRRUS Pathology.^2^ Pathologists were presented with a web view of a slide, where they could navigate the entire slide and inspect the tissue at different magnifications, but without access to the clinical variables. The pathologists could either give a score from 0 to 100 or mark the slide as not scorable. Only slides scored by both pathologists were used for biomarker development and evaluation, the rest was excluded (see Fig. 2). When multiple slides per patient were available, the slide-level scores were averaged to obtain a single case-level score. We refer to the averaged visual score as vTILs.

### Evaluation and statistical analysis

In order to evaluate the predictive performance of the biomarkers for pCR, we calculated the area under the receiver operating characteristic curve (AUC) and performed multivariable logistic regression, always separately for TNBC and Luminal B. The AUC was computed for the NKI development and evaluation sets and the combined RUMC and SCDC cohorts. The provided *p* values were not corrected for multiple testing, since all tested biomarkers are based on validated knowledge of the biology of breast cancer.

The multivariable logistic regression was performed using the NKI evaluation sets only. The RUMC and SCDC cohorts had too small sample sizes and missing clinical information for proper multivariable analysis. All biomarkers were dichotomized based on their median, except for MTR which was dichotomized as 0 or >0, because approximately 60% had a value of 0. The clinical covariates age, grade, T-stage and N-stage were tested as confounding factors. For the MTR biomarker, grade was not tested as confounder, since the mitotic count is part of grading and therefore naturally correlated with grade. For Luminal B, numbers per category were too small in the evaluation set, so no adjusted ORs could be calculated. The covariates were categorized as follows: Age, ≤50 or >50; grade, 2 or 3; T-stage, 1+2 or 3+4; and N-stage, 0 or 1. A covariate was considered a confounder and added to the final multivariable logistic regression model if there was at least 10% change in odds ratio (Exp(B)). The statistical analyses were performed using IBM SPSS Statistical software version 27. The *p* values in the multivariable analysis were determined by Wald test per variable.

## Results

### Tissue segmentation

The segmentation performance was evaluated on the ${\text{dev}}_{\rm seg}^{\rm test}$ via the pixel-wise prediction accuracy using manual annotations as reference standard. On the NKI slides of the test set, the network segmented 93% of annotated tumor and 84% percent of the annotated lymphocytes correctly, while 15% of lymphocytes were wrongly predicted as tumor. The overall accuracy was 95%. Figure 3 (top) shows a segmentation example. On BCSS, 90% of tumor and 56% of lymphocytes were correctly predicted, while 33% of lymphocytes were classified as stroma. The overall accuracy was 76%, which is comparable to the accuracy of 80% reported in the BCSS study [30]. The full normalized confusion matrices are shown in Additional file 1: Fig. S1. Additional segmentation results are shown in Additional file 1: Fig. S2.

The segmentation is the foundation of the PROACTING biomarkers. An example for the determined tumor bulk necessary to compute cTILs is shown in Fig. 4 (top). The tumor bulk for this core consists of four regions, from which the lymphocyte and stroma predictions are counted to compute cTILs. An example for the ITR biomarker is shown in Fig. 4 (bottom), where the 80 μm-radius around segmented lymphocytes is marked with dark ovals.

### Mitosis detection

The mitoses predictions on six NKI TNBC slides were checked by a pathologist (LT). Mitoses predictions outside of tumor regions were filtered out. Without the filtering, the mitoses recall was 98% with precision 32%, while with filtering the recall was 64% with precision of 60%. Filtering removed around 77% of the detected mitoses on the NKI slides, 55% on the SCDC slides and 59% on the RUMC slides. Figure 3 (bottom) shows an example with seven kept and seven removed mitosis detections.

### Biomarker evaluation

**Fig. 5:**
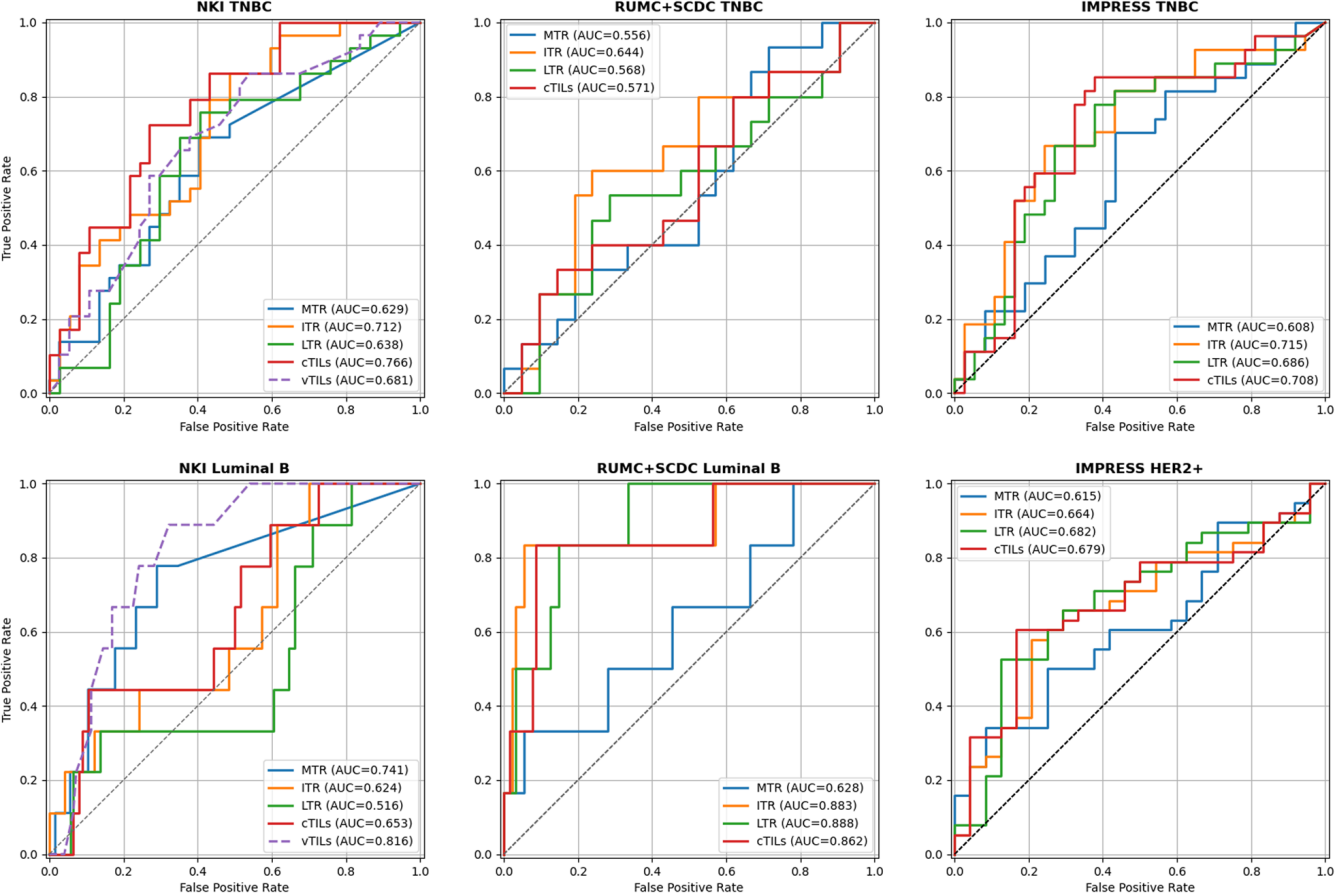
Receiver Operating Characteristic (ROC) curves for predicting pCR on the evaluation sets

*Individual biomarkers* The AUC results for pCR prediction on all cohorts are listed in Tables 3 and 4. The ROC-curves for the biomarkers stratified per cancer molecular subtype on the evaluation set are shown in Fig. 5.

**Table 3:** Evaluation results: AUCs, *p* values and confidence intervals for predicting pCR for each biomarker and cohort. *N* and ${N}_{\text {pcr}}$ are the number of cases and responders, respectively

Biomarker	AUC	*p*	95% CI
NKI TNBC dev_bm_ *N* = 76 (*N*_pcr_ = 41)			
vTILs	0.551	0.447	0.421–0.681
cTILs	0.479	0.759	0.344–0.615
LTR	0.546	0.488	0.414–0.679
ITR	0.518	0.782	0.383–0.654
MTR	0.494	0.929	0.362–0.626
NKI TNBC val_int_ *N* = 66 (*N*_pcr_ = 29)			
vTILs	0.681	0.012	0.551–0.810
cTILs	0.766	0.000	0.653–0.879
LTR	0.638	0.055	0.502–0.775
ITR	0.712	0.003	0.589–0.835
MTR	0.629	0.074	0.493–0.765
RUMC+SCDC TNBC val_int_ *N* = 36 (*N*_pcr_ = 15)			
cTILs	0.571	0.470	0.378–0.765
LTR	0.568	0.490	0.372–0.764
ITR	0.644	0.144	0.456–0.833
MTR	0.556	0.574	0.364–0.747
NKI Luminal B dev_bm_ *N* = 276 (*N*_pcr_ = 28)			
vTILs	0.739	0.002	0.715–0.918
cTILs	0.651	0.009	0.529–0.773
LTR	0.651	0.009	0.531–0.770
ITR	0.665	0.004	0.549–0.782
MTR	0.652	0.008	0.537–0.767
NKI Luminal B dev_bm_ with tumor ≥ 8 mm^2^ *N* = 73 (*N*_pcr_ = 9)			
vTILs	0.896	0.000	0.803–0.989
cTILs	0.731	0.026	0.525–0.937
LTR	0.748	0.016	0.557–0.940
ITR	0.719	0.035	0.514–0.924
MTR	0.696	0.099	0.503–0.890
NKI Luminal B val_int_ *N* = 133 (*N*_pcr_ = 9)			
vTILs	0.816	0.002	0.715–0.918
cTILs	0.653	0.126	0.483–0.823
LTR	0.516	0.872	0.320–0.712
ITR	0.624	0.216	0.445–0.802
MTR	0.741	0.016	0.570–0.912
RUMC+SCDC Luminal B val_int_ *N* = 134 (*N*_pcr_ = 6)			
cTILs	0.862	0.003	0.704–1.000
LTR	0.888	0.001	0.790–0.986
ITR	0.883	0.002	0.718–1.000
MTR	0.628	0.292	0.390–0.865

RUMC+SCDC are the combined RUMC and SCDC datasets

**Table 4:** External evaluation results on the IMPRESS [24] dataset (${\text{val}}_{\rm ext}$): AUCs, *p* values and confidence intervals for predicting pCR

Biomarker	AUC	*p*	95% CI
TNBC *N* = 64 (*N*_pcr_ = 27)			
IMPRESS (H&E only)	0.698	–	–
cTILs	0.708	0.005	0.574–0.841
LTR	0.686	0.012	0.550–0.821
ITR	0.715	0.004	0.584–0.845
MTR	0.608	0.144	0.468–0.747
HER2+ *N* = 62 (*N*_pcr_ = 38)			
IMPRESS (H&E only)	0.812	–	–
cTILs	0.679	0.018	0.543–0.814
LTR	0.682	0.016	0.544–0.820
ITR	0.664	0.016	0.527–0.802
MTR	0.615	0.129	0.475–0.756

All biomarkers have a low performance on the TNBC ${\text{dev}}_{\rm bm}$ data, but cTILs and ITR achieve statistically significant results on the NKI ${\text{val}}_{\rm int}$ set. The TNBC results for RUMC and SCDC combined (RUMC+SCDC) are not statistically significant. On the IMPRESS TNBC ${\text{val}}_{\rm ext}$ set, all TIL-biomarkers achieve statistically significant performance similar or slightly better than the original IMPRESS results.

For Luminal B, ITR exhibits the best performance on the NKI ${\text{dev}}_{\rm bm}$ set and MTR on the ${\text{val}}_{\rm int}$ set. On the RUMC+SCDC cases, all biomarkers except MTR reach relatively high scores; however, the number of cases and responders is small. The relatively low performance of MTR on the RUMC+SCDC cases might be connected to the SCDC slides lower resolution and therefore possibly suboptimal mitoses detection performance. On the IMPRESS HER2+ ${\text{val}}_{\rm ext}$ set, all TIL-biomarkers achieve statistically significant performance below the original IMPRESS results.

Each evaluated biomarker has shown statistically significant performance for at least one data subset, but no biomarker has achieved statistically significant performance on all data subsets. On the external IMPRESS datasets, all computational, TIL-related biomarkers achieve statistically significant performance, showing generalization to fully external data and a different breast cancer subtype (HER2+). MTR achieves high performance on NKI Luminal B, but its results are not significant for TNBC and HER2+. LTR performs similar or below cTILs and ITR, which have similar performance. Overall, for Luminal B, none of the computational biomarkers reach the performance of the visual TILs-score, while for TNBC cTILs and ITR perform slightly better with only small differences in their performance. A definite comparison, however, is not possible due to the relatively small number of evaluation cases.

*Influence of the tumor amount on the prediction* The biopsy slides vary in size and (predicted) tumor amount. The TNBC cases from NKI, SCDC and RUMC have median tumor area of 3.7 mm^2^, 15.3 mm^2^ and 43.6 mm^2^, and the Luminal B cases median tumor area of 4.5 mm^2^, 8.6 mm^2^ and 49.6 mm^2^, respectively (combining all slides per case). This raises the question, whether the difference in the available tumor amount has an influence on the predictive performance. To this effect, we investigated the NKI Luminal B ${\text{dev}}_{\rm bm}$ set. First, we verified that the tumor amount itself is not a predictor: The absolute (predicted) tumor-area as biomarker reaches only an AUC of 0.577 with ${p}=0.18$. Next, we evaluated the predictive performance on the 72 NKI Luminal B ${\text{dev}}_{\rm bm}$ cases with at least 8 mm^2^ of tumor. On this subset, the performance of all biomarkers is increased compared to the full ${\text{dev}}_{\rm bm}$ set (cmp. Table 3). A similar selection from the NKI TNBC ${\text{dev}}_{\rm bm}$ set yielded only 20 cases and no significant biomarker performance (see Additional file 1: Section S1.2 and Fig. S3 for more details).

*Multivariable regression analysis* The statistical results for the logistic multivariable analysis for the NKI TNBC and Luminal B evaluation cohorts are shown in Tables 5 and 6, respectively. For NKI Luminal B, however, the number of responders was too small to run an adjusted model. For NKI TNBC, the only statistically significant biomarker in the multivariable analysis was LTR (OR 4.57, 95% CI 1.29–16.18; *p* = 0.019); vTILs showed a trend toward significance (OR 2.58, 95% CI 0.85–7.87; *p* = 0.095).

**Table 5:** NKI TNBC evaluation set (part of ${\text{val}}_{\rm int}$) multivariable analysis results - unadjusted with only the analyzed biomarker and adjusted together with the clinical variables age, grade, T-Stage and N-Stage

Biomarker	Unadjusted			Adjusted		
	OR	95% CI	*p* value	OR	95%CI	*p* value
vTILs	3.65	1.30–10.22	0.014	2.58*	0.85–7.87	0.095
cTILs	5.03	1.66–15.25	0.004	2.37’	0.69–8.09	0.169
LTR	4.61	1.57–13.50	0.005	4.57’	1.29–16.18	0.019
ITR	2.79	1.02–7.64	0.046	1.70’	0.53–5.48	0.373
MTR	2.23	0.82–6.08	0.116	nc	nc	nc

*adjusted for grade, ’adjusted for grade and T-stage. nc: no change

**Table 6:** NKI Luminal B evaluation set (part of ${\text{val}}_{\rm int}$) multivariable analysis results - unadjusted with only the analyzed biomarker

Biomarker	Unadjusted			Adjusted		
	OR	95%CI	*p* value	OR	95%CI	*p* value
vTILs	10.04	1.22–82–69	0.032	–	–	–
cTILs	3.18	0.64–15.91	0.160	–	–	–
LTR	0.43	0.10–1.78	0.241	–	–	–
ITR	0.93	0.24–3.36	0.921	–	–	–
MTR	6.59	1.31–33.13	0.022	–	–	–

No adjusted results because of too small number of events

*Visual TILs* The pathologist’ vTILs biomarker achieves the highest performance on both the NKI Luminal B ${\text{dev}}_{\rm bm}$ and evaluation set and a relatively good performance on the NKI TNBC evaluation set. In comparison, cTILs achieves higher performance on the NKI TNBC evaluation set, but not on Luminal B. The computational biomarker with the highest Spearman correlation with vTILs is cTILs with a correlation of 0.78 for NKI TNBC and 0.57 for NKI Luminal B. The correlation between the individual pathologist vTILs is 0.68 in both cohorts. On the ${\text{dev}}_{\rm bm}$ sets, there is no difference in the performance of the individual pathologist visual scores for NKI TNBC, but for NKI Luminal B the individual pathologist scores achieve AUCs of 0.684 and 0.775. This underlines the subjective manner of TIL-scoring, which can have a strong effect on predictive performance.

## Discussion

In this work, we presented a set of computational biomarkers solely based on routinely available H&E stained slides, to predict response to NAC. In the literature, a wide range of computational, deep-learning-based biomarkers for pCR prediction have been recently proposed [20–24]. However, they usually lack interpretability as they tend to be based on end-to-end learning from raw data to outcome prediction. Our approach, based on hypothesis-driven biomarkers, encodes measurable aspects of the TME based on simple formulas, which could potentially be applied also when tissue and cell quantification is performed using computer algorithms different from ours, as well as using semi-automatic or fully visual estimation by pathologists (e.g., mitotic count, manual tumor segmentation, etc.). In this sense, the proposed approach makes biomarker quantification largely applicable. Furthermore, the external validation performed on a publicly available dataset, as well as the public release of PROACTING segmentation-based biomarkers, makes comparison with our results possible for the research community.

The PROACTING biomarkers exhibit different performances on different subsets of the data with no clear prevalence of a single biomarker. This is partly due to the relative small sample sizes analyzed per molecular subtype and center, diminishing predictive power. For this reason, although biomarkers showed significant predictive value in some cohorts, we cannot refer to proper absolute statistical significance for any of the considered biomarkers. While the data used in this work revealed not to be sufficient to definitely prove benefit for patient care, we believe it presents evidence to further pursue automated TIL scoring for clinical outcome prediction and treatment planning, and to further investigate the role of mitotic count as a predictive biomarker.

ROC analysis showed that the cTILs biomarker (see Fig. 5), although not reaching statistical significance, achieved 100% sensitivity in the NKI cohort, both for TNBC and Luminal B, with a similar behavior in the RUMC + SCDC Luminal B cohort. If confirmed on larger validation studies, this effect might indicate a potential value of this biomarker in clinical applications, especially when we consider the current clinical scenario, where most breast cancer patients are treated with neoadjuvant chemotherapy, while a substantial fraction of them do not respond. Reaching 100% sensitivity indicates the potential possibility of detecting all responders at the cost of treating a fraction of non-responders, instead of de-facto treating all patients. This characteristics could lead to potential reduction of overtreatment in future. Furthermore, the computational PROACTING biomarkers rely exclusively on H&E staining, the most commonly accessible staining technique in histopathology. While more precise predictions with IHC staining may be possible [24], its routine availability is often limited. Consequently, the developed computational biomarkers hold broad and more direct applicability to clinical practice.

The applicability of the models may be further improved by considering the available tumor tissue amount, which varies due to differences in the number of cores on the slide and their sizes. Slides with small tumor regions might not be representative for the whole tumor and therefore do not contain enough information for a reliable prediction. Small tumor amounts might also enhance the effect of suboptimal segmentation performance on the computed biomarkers and lead to deteriorated predictive performance. Therefore, evaluation on large, multi-centric external cohorts is required to reliably verify these results. The observed increase of the predictive performance for Luminal B on slides with relative high amounts of segmented tumor indicates that a more reliable pCR prediction is possible, even if only for a subset of cases.

TILs scoring can be interpreted as measuring the lymphocyte density within the stroma in the tumor-bulk. An important question is, therefore, how to determine the tumor-bulk in biopsies, where less tissue is available and small changes in the definition can have a large impact on the score. Specifically of interest is the scenario when several close-by tumor regions surrounded by lymphocytes have stroma in-between. If such tumor regions are scored individually, the averaged score would be high. However, if the stroma in-between is included, the resulting TILs-score would be low. A large clustering-distance, such as the 750 microns proposed for resections [18], might result in most of the core being included in the tumor bulk. For example, in Fig. 4 (top), it would lead to the four tumor-regions being merged into a single encompassing tumor-bulk with a much increased stroma content and only marginally increased lymphocyte content resulting in a lower score. To avoid such ‘under-scoring’ in biopsies, we chose smaller distances and margins in this work. Determining the appropriate settings for biopsies, perhaps also taking the morphological type into consideration, might be required for more stable TIL-scoring, both by pathologists and computationally.

Our trained segmentation model has two limitations that might affect biomarker performance. First, it does not differentiate between invasive and in situ cancer on the assumption that the amount of in situ tumor co-occurring with invasive tumor would be negligible in core-needle biopsies. Explicitly classifying and excluding in situ tumor, however, as suggested for TIL-scoring on surgical resections [6], might yield a more fine-grained biomarker performance.

Second, the segmentation model seems sometimes to miss single or sparsely occurring lymphocytes (see Fig. 3, where single lymphocytes below the red lymphocyte annotation are not segmented). This is probably due to most of the annotated lymphocytes being from clusters of lymphocytes, as these are easier to recognize and annotate. Being able to recognize single lymphocytes would enable more fine-grained biomarkers at the cost of gathering sufficient training data. It is, however, unclear, whether this would improve biomarker performance, since isolated lymphocytes will not contribute substantially to the TILs quantification. In future, these limitations can be addressed by retraining or fine-tuning the segmentation model. Our two-step approach’s modularity allows for the replacement of the segmentation model with another (as long as it also predicts tumor, stroma, and lymphocytes) or even manual segmentation, all without altering the entire approach. This sets our approach apart from ‘end-to-end’ deep learning methods, as it is both easier to interpret and maintain in practice.

## Conclusion

The study evaluates multiple computational biomarkers and validates computational TILs as effective biomarkers with predictive capability while eliminating subjective scoring bias present in manual TIL scoring. Similarly, automating the assessment of mitotic count also holds predictive potential, albeit with a slightly lower predictive performance. Predicting pCR is both highly clinically relevant and challenging, as it is currently unknown if the pre-treatment biopsies contain sufficient information for a reliable prediction in clinical routine. Additional factors like the small biopsy sizes and staining artifacts further increase the difficulty level. Nevertheless, we could achieve predictive performance with our computational biomarkers while maintaining morphological interpretability, confirming their predictive value via external independent validation. We were able to reach AUCs in the range 0.66–0.88 depending on the cancer subtype and center. These results show that reliable pCR prediction might be possible, even if only for a subset of cases, potentially allowing automated, reproducible identification of patients at risk of over-treatment. We also evaluated the predictive value of the automated mitotic count from routine H&E biopsy slides adding to the knowledge on tumor proliferation in the context of neoadjuvant chemotherapy response. Further research will involve validation of the presented techniques in larger cohorts.

### Supplementary Information

**Additional file 1.** Supplementary results for the evaluation of the segmentation model, and analysis of the influence of the tumor amount.

## Data Availability

We made algorithms to compute ITR, LTR and cTILs publicly available on the grand-challenge.org platform, which can be accessed upon request for research purposes at https://grand-challenge.org/algorithms/bc-seg-det-rumc. Data from the TCGA-BRCA cohort used to train the segmentation model were derived from the BCSS project and can be accessed at https://bcsegmentation.grand-challenge.org. The IMPRESS data are available at https://tinyurl.com/IMPRESS-DATA.

## Footnotes

1 https://www.slidescore.com.

2 https://grand-challenge.org/.

## References

[1] Masood S (2016). Neoadjuvant chemotherapy in breast cancers. Womens Health.

[2] Asaoka M, Gandhi S, Ishikawa T, Takabe K (2020). Neoadjuvant chemotherapy for breast cancer: past, present, and future. Breast Cancer: Basic Clin Res.

[3] Gamucci T, Pizzuti L, Sperduti I, Mentuccia L, Vaccaro A, Moscetti L, Marchetti P, Carbognin L, Michelotti A, Iezzi L (2018). Neoadjuvant chemotherapy in triple-negative breast cancer: a multicentric retrospective observational study in real-life setting. J Cell Physiol.

[4] Bonnefoi H, Litière S, Piccart M, MacGrogan G, Fumoleau P, Brain E, Petit T, Rouanet P, Jassem J, Moldovan C (2014). Pathological complete response after neoadjuvant chemotherapy is an independent predictive factor irrespective of simplified breast cancer intrinsic subtypes: a landmark and two-step approach analyses from the eortc 10994/big 1–00 phase iii trial. Ann Oncol.

[5] Denkert C, Loibl S, Noske A, Roller M, Muller B, Komor M, Budczies J, Darb-Esfahani S, Kronenwett R, Hanusch C (2010). Tumor-associated lymphocytes as an independent predictor of response to neoadjuvant chemotherapy in breast cancer. J Clin Oncol.

[6] Salgado R, Denkert C, Demaria S, Sirtaine N, Klauschen F, Pruneri G, Wienert S, Van den Eynden G, Baehner FL, Pénault-Llorca F (2015). The evaluation of tumor-infiltrating lymphocytes (tils) in breast cancer: recommendations by an international tils working group 2014. Ann Oncol.

[7] Denkert C, von Minckwitz G, Darb-Esfahani S, Lederer B, Heppner BI, Weber KE, Budczies J, Huober J, Klauschen F, Furlanetto J (2018). Tumour-infiltrating lymphocytes and prognosis in different subtypes of breast cancer: a pooled analysis of 3771 patients treated with neoadjuvant therapy. Lancet Oncol.

[8] Gao G, Wang Z, Qu X, Zhang Z (2020). Prognostic value of tumor-infiltrating lymphocytes in patients with triple-negative breast cancer: a systematic review and meta-analysis. BMC Cancer.

[9] Kos Z, Roblin E, Kim RS, Michiels S, Gallas BD, Chen W, van de Vijver KK, Goel S, Adams S, Demaria S (2020). Pitfalls in assessing stromal tumor infiltrating lymphocytes (stils) in breast cancer. NPJ Breast Cancer.

[10] Gerdes J, Li L, Schlueter C, Duchrow M, Wohlenberg C, Gerlach C, Stahmer I, Kloth S, Brandt E, Flad H (1991). Immunobiochemical and molecular biologic characterization of the cell proliferation-associated nuclear antigen that is defined by monoclonal antibody ki-67. Am J Pathol.

[11] Tao M, Chen S, Zhang X, Zhou Q (2017). Ki-67 labeling index is a predictive marker for a pathological complete response to neoadjuvant chemotherapy in breast cancer: a meta-analysis. Medicine.

[12] Urruticoechea A, Smith IE, Dowsett M (2005). Proliferation marker ki-67 in early breast cancer. J Clin Oncol.

[13] Russakovsky O, Deng J, Su H, Krause J, Satheesh S, Ma S, Huang Z, Karpathy A, Khosla A, Bernstein M (2015). Imagenet large scale visual recognition challenge. Int J Comput Vis.

[14] Van der Laak J, Litjens G, Ciompi F (2021). Deep learning in histopathology: the path to the clinic. Nat Med.

[15] Saltz J, Gupta R, Hou L, Kurc T, Singh P, Nguyen V, Samaras D, Shroyer KR, Zhao T, Batiste R (2018). Spatial organization and molecular correlation of tumor-infiltrating lymphocytes using deep learning on pathology images. Cell Rep.

[16] Amgad M, Sarkar A, Srinivas C, Redman R, Ratra S, Bechert CJ, Calhoun BC, Mrazeck K, Kurkure U, Cooper LA, et al. Joint region and nucleus segmentation for characterization of tumor infiltrating lymphocytes in breast cancer. In: Medical Imaging 2019: Digital Pathology, 2019; vol. 10956, pp. 129–136. SPIE. 10.1117/12.2512892 PMC6988758 31997849

[17] Le H, Gupta R, Hou L, Abousamra S, Fassler D, Torre-Healy L, Moffitt RA, Kurc T, Samaras D, Batiste R (2020). Utilizing automated breast cancer detection to identify spatial distributions of tumor-infiltrating lymphocytes in invasive breast cancer. Am J Pathol.

[18] Thagaard J, Stovgaard ES, Vognsen LG, Hauberg S, Dahl A, Ebstrup T, Doré J, Vincentz RE, Jepsen RK, Roslind A (2021). Automated quantification of stil density with H&E-based digital image analysis has prognostic potential in triple-negative breast cancers. Cancers.

[19] Amgad M, Salgado R, Cooper LA. Mutils: explainable, multiresolution computational scoring of tumor-infiltrating lymphocytes in breast carcinomas using clinical guidelines. medRxiv 2022.

[20] Li F, Yang Y, Wei Y, He P, Chen J, Zheng Z, Bu H (2021). Deep learning-based predictive biomarker of pathological complete response to neoadjuvant chemotherapy from histological images in breast cancer. J Transl Med.

[21] Saednia K, Lagree A, Alera MA, Fleshner L, Shiner A, Law E, Law B, Dodington DW, Lu F-I, Tran WT (2022). Quantitative digital histopathology and machine learning to predict pathological complete response to chemotherapy in breast cancer patients using pre-treatment tumor biopsies. Sci Rep.

[22] Li F, Yang Y, Wei Y, Zhao Y, Fu J, Xiao X, Zheng Z, Bu H (2022). Predicting neoadjuvant chemotherapy benefit using deep learning from stromal histology in breast cancer. NPJ Breast Cancer.

[23] Duanmu H, Bhattarai S, Li H, Shi Z, Wang F, Teodoro G, Gogineni K, Subhedar P, Kiraz U, Janssen EAM, Aneja R, Kong J (2022). A spatial attention guided deep learning system for prediction of pathological complete response using breast cancer histopathology images. Bioinformatics.

[24] Huang Z, Shao W, Han Z, Alkashash AM, De la Sancha C, Parwani AV, Nitta H, Hou Y, Wang T, Salama P (2023). Artificial intelligence reveals features associated with breast cancer neoadjuvant chemotherapy responses from multi-stain histopathologic images. NPJ Precis Oncol.

[25] Ogier du Terrail J, Leopold A, Joly C, Béguier C, Andreux M, Maussion C, Schmauch B, Tramel EW, Bendjebbar E, Zaslavskiy M (2023). Federated learning for predicting histological response to neoadjuvant chemotherapy in triple-negative breast cancer. Nat Med.

[26] Cortazar P, Zhang L, Untch M, Mehta K, Costantino JP, Wolmark N, Bonnefoi H, Cameron D, Gianni L, Valagussa P (2014). Pathological complete response and long-term clinical benefit in breast cancer: the ctneobc pooled analysis. Lancet.

[27] Lips E, Mulder L, De Ronde J, Mandjes I, Koolen B, Wessels L, Rodenhuis S, Wesseling J (2013). Breast cancer subtyping by immunohistochemistry and histological grade outperforms breast cancer intrinsic subtypes in predicting neoadjuvant chemotherapy response. Breast Cancer Res Treat.

[28] Tellez D, Balkenhol M, Otte-Höller I, van de Loo R, Vogels R, Bult P, Wauters C, Vreuls W, Mol S, Karssemeijer N (2018). Whole-slide mitosis detection in H&E breast histology using phh3 as a reference to train distilled stain-invariant convolutional networks. IEEE Trans Med Imaging.

[29] Balkenhol MC, Bult P, Tellez D, Vreuls W, Clahsen PC, Ciompi F, van der Laak JA (2019). Deep learning and manual assessment show that the absolute mitotic count does not contain prognostic information in triple negative breast cancer. Cell Oncol.

[30] Amgad M, Elfandy H, Hussein H, Atteya LA, Elsebaie MA, Abo Elnasr LS, Sakr RA, Salem HS, Ismail AF, Saad AM (2019). Structured crowdsourcing enables convolutional segmentation of histology images. Bioinformatics.

[31] Weinstein JN, Collisson EA, Mills GB, Shaw KRM, Ozenberger BA, Ellrott K, Shmulevich I, Sander C, Stuart JM, Network CGAR (2013). The cancer genome atlas pan-cancer analysis project. Nat Genet.

[32] van Rijthoven M, Balkenhol M, Siliņa K, van der Laak J, Ciompi F (2021). Hooknet: multi-resolution convolutional neural networks for semantic segmentation in histopathology whole-slide images. Med Image Anal.

[33] Ronneberger O, Fischer P, Brox T. U-Net: Convolutional networks for biomedical image segmentation. 2015;9351:234–41.

[34] Bándi P, Balkenhol M, van Ginneken B, van der Laak J, Litjens G (2019). Resolution-agnostic tissue segmentation in whole-slide histopathology images with convolutional neural networks. PeerJ.

