# Supplementary material for: PROACTING: predicting pathological complete response to neoadjuvant chemotherapy in breast cancer from routine diagnostic histopathology biopsies with deep learning
Source: Breast Cancer Res. 2023 Nov 13;25:142. doi: 10.1186/s13058-023-01726-0 (PMC10644597; doi:10.1186/s13058-023-01726-0)
Supplement: Supplementary file 1 — Additional file 1. Supplementary results for the evaluation of the segmentation model, and analysis of the influence of the tumor amount. [file 13058_2023_1726_MOESM1_ESM.pdf]

Supplementary material to: PROACTING: Predicting pathological complete response to neoadjuvant chemotherapy in breast cancer from routine diagnostic histopathology biopsies with deep learning

September 3, 2023

Contents

S1 Supplementary Appendix **1**
S1.1 Segmentation Model 1
S1.2 Influence of the tumor amount on the prediction 4

List of Figures

S1 Confusion matrix of the segmentation model 2
S2 Visual examples of two breast cancer biopsies from the test set . 3
S3 Evaluation on the NKI Luminal B development set with different tumor amount cutoffs 5

S1 Supplementary Appendix

S1.1 Segmentation Model

In our U-Net implementation we used a depth of four and in the decoder-part bilinear upsampling followed by 2x2 convolutions. We trained the network on patches of size 412x412 px at 20x magnification ($0.5\mu m/px$ spacing) with the patch data-augmentations flip, rotate, scale, color, contrast and noise. The network was trained with learning rate decay starting from learning rate 0.0005 reduced by half after 15 epochs without improvement in validation accuracy. The training was stopped early if there was no improvement after 30 epochs. In

each epoch 19.200 patches were sampled with class-wise balanced sampling: each tissue class had the same sampling probability except for fatty tissue, which was sampled five times less frequent.

Fig. S1 shows the confusion matrix on the NKI and BCSS test sets. Fig. S2 shows two segmentation examples.

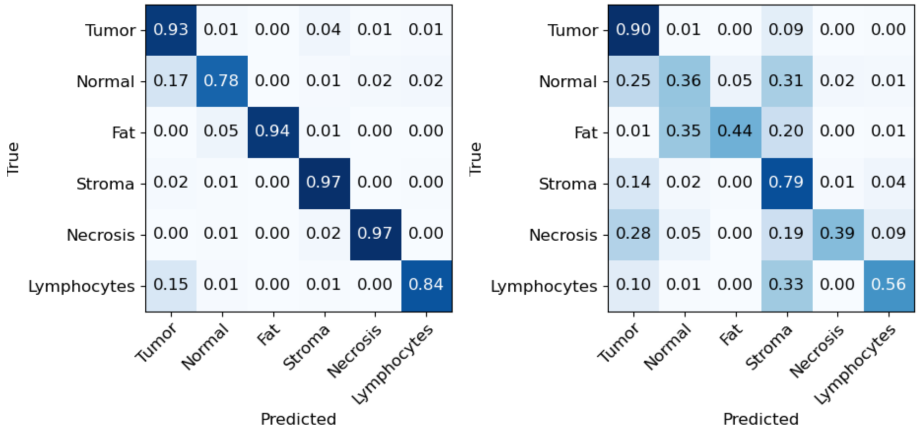

Figure S1: Normalized confusion matrix of the segmentation model for the 15 NKI test slides (left) and 59 TCGA slides (right). It shows in each cell the ratio of the (true) class on the y-axis predicted as the class on the x-axis, computed on the pixel-level.

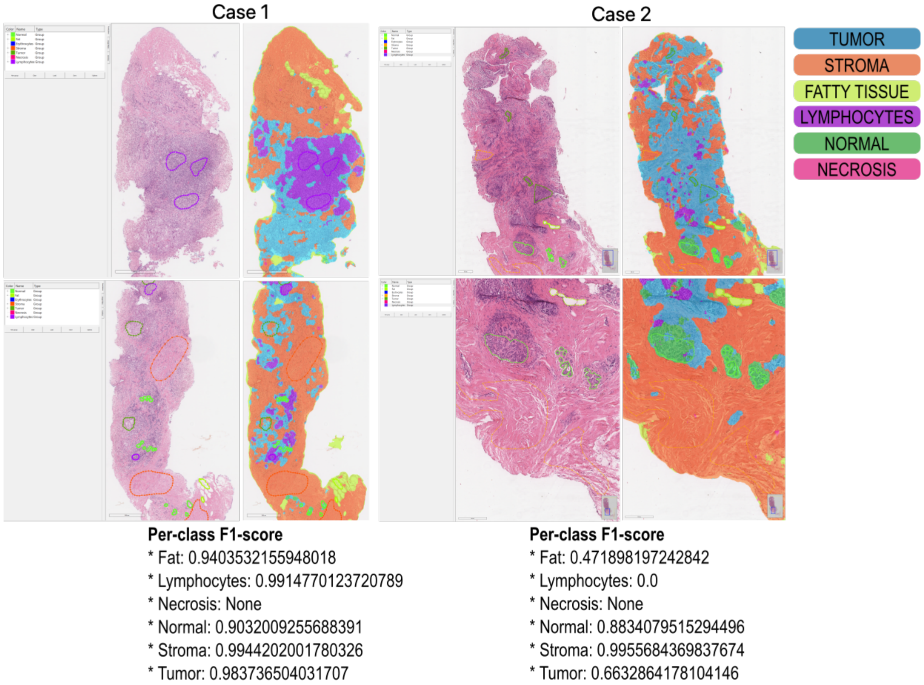

Figure S2: Visual examples of two breast cancer biopsies from the test set. For each case, we show on the left the H&E slide visualized using the ASAP software with manual annotations of sparse regions (polygons) performed by pathologists, with color coding visible in the ASAP annotation plug-in; on the right we show the segmentation output from our segmentation model. The color legend is shown on the right. The top part of each case shows the top part of the biopsy, the bottom part shows a zoomed-in part of the bottom of the biopsy. On case 1, our segmentation model yielded a performance with F1-score > 0.9 in all annotated classes. On case 2, a lower performance was obtained for the classes fat, normal and tumor.

S1.2 Influence of the tumor amount on the prediction

We also investigated how the segmented tumor amount affects the performance of the biomarkers. Its distribution in the cohorts is visualized in Fig. S3a and its influence is depicted in Fig. S3b and S3c, which show the evaluation of the biomarkers on the NKI development sets partitioned to subsequently exclude cases with less then the given minimal tumor area in $\mathbf{mm^2}$. This visualization reveals a trend for Luminal B: The pCR prediction improves with increased amount of (predicted) tumor, especially for vTILs.

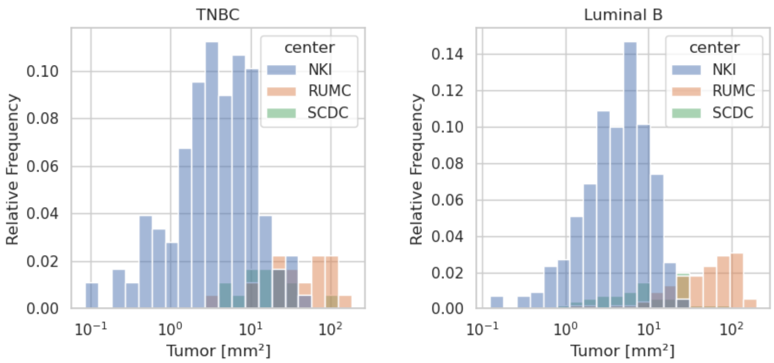

(a) Segmented tumor area histogram

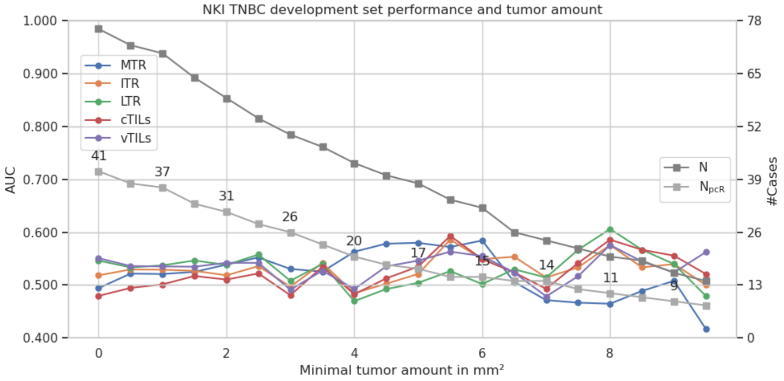

(b) NKI TNBC tumor cutoff evaluation

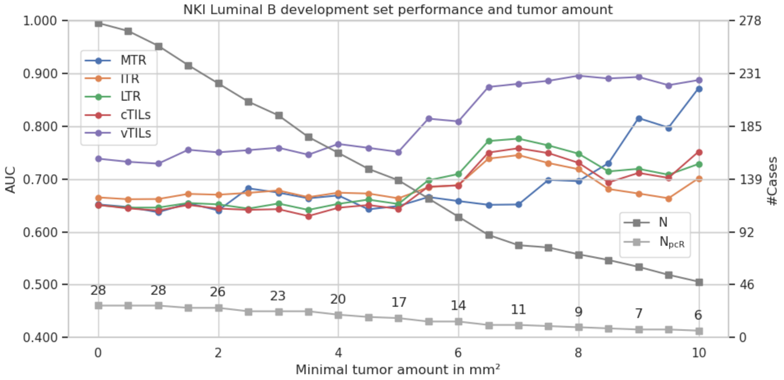

(c) Luminal B tumor cutoff evaluation

Figure S3: Histogram of the segmented tumor (top) and Biomarker-AUC for pCR evaluated with different (minimal) tumor amount cutoffs on the NKI development set. N and N_{pCR} are the overall number of cases and responders after the cutoff. The 0mm^2 cutoff corresponds to the evaluation of all cases. At the 12mm^2 cutoff only 30 cases with 3 responders are left.
